# Disrespect and abuse as a predictor of postnatal care utilisation and maternal-newborn well-being: a mixed-methods systematic review

**DOI:** 10.1136/bmjgh-2020-004698

**Published:** 2021-04-21

**Authors:** Nicole Minckas, Lu Gram, Colette Smith, Jenevieve Mannell

**Affiliations:** Institute for Global Health, University College London, London, UK

**Keywords:** maternal health, obstetrics, health services research, systematic review

## Abstract

**Introduction:**

Globally, a substantial number of women experience abusive and disrespectful care from health providers during childbirth. As evidence mounts on the nature and frequency of disrespect and abuse (D&A), little is known about the consequences of a negative experience of care on health and well-being of women and newborns. This review summarises available evidence on the associations of D&A of mother and newborns during childbirth and the immediate postnatal period (understood as the first 24 hours from birth) with maternal and neonatal postnatal care (PNC) utilisation, newborn feeding practices, newborn weight gain and maternal mental health.

**Methods:**

We conducted a systematic review of all published qualitative, quantitative and mixed-methods studies on D&A and its postnatal consequences across all countries. Pubmed, Embase, Web of Science, LILACS and Scopus were searched using predetermined search terms. Quantitative and qualitative data were analysed and presented separately. Thematic analysis was used to synthesise the qualitative evidence.

**Results:**

A total of 4 quantitative, 1 mixed-methods and 16 qualitative studies were included. Quantitative studies suggested associations between several domains of D&A and use of PNC as well as maternal mental health. Different definitions of exposure meant formal meta-analysis was not possible. Three main themes emerged from the qualitative findings associated with PNC utilisation: (1) women’s direct experiences; (2) women’s expectations and (3) women’s agency.

**Conclusion:**

This review is the first to examine the postnatal effect of D&A of women and newborns during childbirth. We highlight gaps in research that could help improve health outcomes and protect women and newborns during childbirth. Understanding the health and access consequences of a negative birth experience can help progress the respectful care agenda.

**Key questions**

**What is already known?**

- A substantial number of women experience disrespectful and abusive care (D&A) from health providers during childbirth.
- A study conducted in four low/middle-income countries (LMICs) found that 41.6% of women had experienced one or more episodes of physical abuse, verbal abuse, stigma or discrimination; with younger, poorer, unemployed, illiterate and unmarried women being the most at risk.
- Postnatal care (PNC) use has consistently had among the lowest coverage among interventions on the continuum of maternal and childcare, with a reported median coverage for the 75 LMICs in the Countdown to 2030 initiative at 28% for babies and 58% for mothers.
- A recent qualitative study showed that factors affecting utilisation of PNC not only include cost and distance to the healthcare facility and lack of knowledge of the importance of PNC, but also fear of mistreatment by healthcare workers, fear of denial of PNC or actual denial or delay of care.

**What are the new findings?**

- Our systematic review shows that experiencing D&A during childbirth is associated with reduced utilisation of maternal or neonatal PNC, particularly when the woman did not have a companion at the birth, was not offered a choice of birth position or when she perceived that the facility was not clean.
- Women’s decision to seek PNC is not solely influenced by their previous experience but also by other factors such as their own expectations and agency which are shaped by broader cultural, social and gender norms.
- Experiencing verbal abuse from health providers during childbirth can also take a toll on women’s mental health, increasing the likelihood of developing depression during the postpartum period.

**Key questions**

**What do the new findings imply?**

- Health providers and policymakers should increase efforts to guarantee high-quality, respectful, dignified and supportive care throughout the continuum of care to increase coverage of essential services and to promote and protect the health and well-being of women and newborns.
- Our findings shed light on the potential consequences of D&A on healthcare seeking behaviours; more research is urgently needed to understand the public health implications of a negative experience at birth on the health of women and newborns.

## Introduction

During the millennium development goal era, global efforts were aimed at ensuring that all women and their newborns had access to skilled care before, during and after childbirth as a way to reduce maternal and neonatal morbidity and mortality.^1^ Despite achieving a 45% reduction in the maternal mortality ratio between 1990 and 2013, these efforts fell short of the target.^2^ In recent years, evidence emerged suggesting that the quality of care received by women and newborns during facility-based birth was not meeting the required standard.^3–7^ Around the world, a substantial number of women experienced abusive and disrespectful care from health providers.

In 2010, the publication of the landscape analysis on disrespect and abuse (D&A) by Bowser and Hill led to the global recognition of the poor treatment women experienced during childbirth, including physical abuse, non-consented care or discrimination by healthcare providers.^3^ Later, a mixed-methods review conducted by Bohren *et al* concluded that ‘D&A’ should be replaced by ‘mistreatment during childbirth’ (MDC), a term that further separates the issue from individual intentionality and links it to the realm of healthcare quality, introducing an alternative 7-domain typology which integrated health systems constraints (box 1). Using the MDC typology, a recent study found that 41.6% of women attending urban maternity hospitals across four low/middle-income countries (LMICs) had experienced at least one episode of physical abuse, verbal abuse, stigma or discrimination.^8^ Younger, poorer, unemployed, illiterate and unmarried women were more likely to experience this type of treatment.^9^

Box 1 **Defining terminology**

For the purpose of this review, we conceptualise ‘disrespect and abuse’ (D&A) as a broad term which encompasses an interpersonal and a structural component. The interpersonal component refers to actions by health providers towards women during childbirth that involve violence, discrimination, oppression or disrespect, regardless of intentionality. The structural component refers to constraints in the health system that affect the provision of good quality care rooted in power asymmetries, institutional structures, economic inequality, or social and gender norms. *Mistreatment during childbirth* overlaps with D&A by referring to instances of poor-quality care at the patient-provider level caused by constraints in the health system. While we recognise more nuances exist between terminologies, we have decided to use D&A as a broader term throughout the text to increase consistency and clarity.

As global evidence mounts on the nature and frequency of D&A during childbirth,^10–14^ little is known about the consequences of negative experiences of care on the health and well-being of women and newborns. Negative experiences during antenatal, intrapartum or immediate postpartum care might influence women’s care-seeking behaviour after birth, particularly regarding accessing postnatal care (PNC). A recent qualitative study showed that factors affecting utilisation of PNC not only include cost and distance to the healthcare facility and lack of knowledge of the importance of PNC, but also fear of mistreatment by healthcare workers, fear of denial of PNC or actual denial of care.^15^ This offers a potential hypothesis to explain why, despite great improvements in overall access to institutional care, PNC has consistently had among the lowest coverage on the continuum of maternal and child care.^16 17^ While many of the arguments in favour of preventing D&A are framed in the context of human rights or maternal and neonatal survival, it is also necessary to understand how the childbirth experience can affect the interactions of women and newborns with the healthcare system, and subsequently their health.^18^

PNC includes any provision of healthcare for the woman and the baby during the postnatal period, from childbirth until 6 weeks post partum.^19^ In this period, women and newborns are particularly susceptible to widespread and persistent childbirth-related morbidities, many of which are unreported and go unnoticed and untreated by healthcare professionals.^20^ Common health problems for women include physical morbidity, such as backache,^21 22^ perineal pain,^23 24^ stress incontinence,^25–27^ breastfeeding problems^28–30^ and mental health problems, such as postpartum depression (PPD).^31–34^ The likelihood of depressive episodes after childbirth can be twice as high compared with any other period of a woman’s life.^35^ Women who suffer from postnatal mental health disorders have prolonged difficulties in developing maternal feelings towards their infants compared with women who do not experience these, with direct effects on infants’ health and development, such as delayed psycho-social development, low-birth weight, reduced breast feeding, hampered growth and lower compliance with immunisation schedules.^36 37^ Therefore, timely screening and identification of women’s needs are essential to ensuring women have sufficient support during their initiation to motherhood, to promoting her health and her baby’s and to fostering an environment that offers support to the extended family and community for a wide range of health and social needs.^38–40^ In addition to the need for improvements in the quality of care received by women and newborns in the intrapartum period, evidence on the consequences of D&A on women and newborns’ health, well-being and care-seeking behaviours is necessary to inform programme implementation, policy and advocacy.

This review will answer the following research question: what are the associations of (a) D&A of mother and newborn during childbirth and the immediate postnatal period (understood as the first 24 hours from birth) with (b) maternal and neonatal PNC utilisation, newborn feeding practices, newborn weight gain and maternal mental health?

## Methods

### Type of review

A mixed-methods review was conducted following a parallel-result segregated synthesis design. In this review, quantitative and qualitative data were analysed and presented separately with integration occurring in the discussion.^41^ The rationale for conducting a mixed-methods review was to acknowledge the complexity of the issue of D&A during childbirth. We not only aimed to quantify the relation between D&A and the selected outcomes (quantitative analysis) but also to explore how other factors enable or inhibit this relationship (qualitative analysis).

### Search strategy

Pubmed, Embase, Web of Science, LILACS and Scopus were systematically searched using controlled vocabulary and free-text terms for: (a) D&A or mistreatment of women or newborns during childbirth, (b) maternal, perinatal, neonatal, postnatal health, (c) access to care, (d) breast feeding or PNC utilisation or PPD or infant weight gain (online supplemental appendix 1). The search was restricted to published articles from 20 September 2010 until March 2020. The starting date of the search was selected as it coincides with the date of the publication of Bowser and Hill’s seminal landscape analysis.^3^ Reviews and reference lists from identified articles were hand searched to identify additional studies.

10.1136/bmjgh-2020-004698.supp1 Supplementary data

### Eligibility criteria

For the quantitative analyses, we included studies if: (a) they were primary research conducted using quantitative research designs; (b) the sample included women who have given birth at a health facility; (c) they measured the association of D&A with PNC utilisation after initial discharge, maternal PPD or other mental health outcome, breast feeding or infant weight gain and (d) were conducted in LMICs by the World Bank definition.^42^

For the qualitative analyses, we included studies if they (a) were primary research conducted using qualitative methods; (b) discussed issues related to D&A, and PNC utilisation, maternal PPD or other mental health outcome, breast feeding or infant weight gain and (c) were conducted in LMICs by the World Bank definition.^42^ No inclusion criteria on the study sample’s characteristics were established for the selection of qualitative studies.

For both quantitative and qualitative studies, we did not impose any restrictions regarding the type of D&A, its operationalisation, definition or measurement tools for inclusion. Grey literature, opinion pieces and editorials, dissertations/theses, policy papers, general reports and conference abstracts were excluded. Studies were also excluded if they focused on people with disabilities, refugees or people from conflict-affected settings, or on women or newborns with severe health conditions that require specialised clinical care. Articles in English, Spanish, Portuguese, Greek, Italian and French were included.

### Data extraction and synthesis

The titles and abstracts retrieved were independently screened by two reviewers (NM, AGN). Unclear abstracts were carried forward to the screening stage. The full texts of potentially eligible articles were retrieved and screened against the inclusion criteria. Disagreements were resolved by discussion between reviewers. For both quantitative and qualitative studies, data were extracted on country, study design, sample size and characteristics of the sample (age, place of residence, occupation, gender/sex, education, socioeconomic status, marital status).

For quantitative studies, primary outcomes were extracted according to the type of abuse reported in the article, independently of whether it was aligned to existing D&A or MDC typologies. If the article reported the exposure in its positive form (such as privacy), it was converted to its negative form (ie, lack of privacy) to ensure consistency across the studies and facilitate interpretation of the review’s results. Measures of effects were also transformed to unadjusted ORs if reported differently to allow for comparison between studies (original effect size without transformation can be found in online supplemental appendix 5). A meta-analysis on the association between mistreatment and the main outcomes was not possible because of the small number of articles and large heterogeneity in the definition of both exposure and outcomes. Therefore, results were summarised descriptively. All calculations were done in the statistical software STATA V.14.

Qualitative studies were imported to the software NVivo V.12 for analysis. Articles were analysed using thematic synthesis.^43^ After becoming familiar with the data, two researchers (NM, AGN) independently coded the results section of each study, line by line, to inductively search for emerging themes. First, we identified codes that addressed the following research questions: (a) does D&A affect women’s decision to use PNC? and (b) does D&A affect other outcomes such as breast feeding, infant growth or women’s mental health? No studies were found to answer the latter question, so the analysis only focused on PNC use as an outcome. At this stage, specific codes related to disrespectful or abusive acts towards women emerged as enablers or deterrents of PNC use. During a second round of coding, we explored underlying mechanisms by which D&A could affect PNC use through the following question: ‘*how* does D&A relate to women’s decision to use PNC?’. This approach allowed us to detect broader factors linking D&A and PNC utilisation. Next, we grouped the codes identified into common descriptive themes. From this exercise, the final three themes emerged: (a) women’s direct experiences, (b) women’s expectations, (c) women’s agency.

### Risk of bias (quality) assessment

For the quantitative studies, we used the National Institutes of Health (NIH) Quality Assessment Tool for Observational Cohort and Cross-Sectional Studies.^44^ The quality of qualitative studies was assessed using the Critical Appraisal Skills Programme quality-assessment tool (http://www.casp-uk.net).^45^

Two reviewers independently assessed each study for quality and categorised the studies on ‘high’ (≥75% of applicable criteria), ‘medium’ (50–<75%) or ‘low’ (<50%) quality. Discussions were held to reach consensus (online supplemental appendices 2 and 3). Because there is no current consensus on the role of quality criteria and how it should be applied,^46^ no studies were excluded as a result of the quality assessment. The Confidence in the Evidence from Reviews of Qualitative Research approach was used to assess the confidence of the qualitative finding by assessing methodological limitations, relevance to the review question, coherence and adequacy of data.^47^ The confidence in the evidence was categorised as high, moderate and low.

### Registrations and reporting

This systematic review is reported following the Preferred Reporting Items for Systematic Reviews and Meta-Analyses statement guidelines^48^ and the ENTREQ statement guideline^49^ to enhance transparency in reporting quantitative and qualitative evidence synthesis. The protocol has been prospectively registered and published in PROSPERO: registration CRD42020208916.

## Results

### General overview

**Figure 1:**
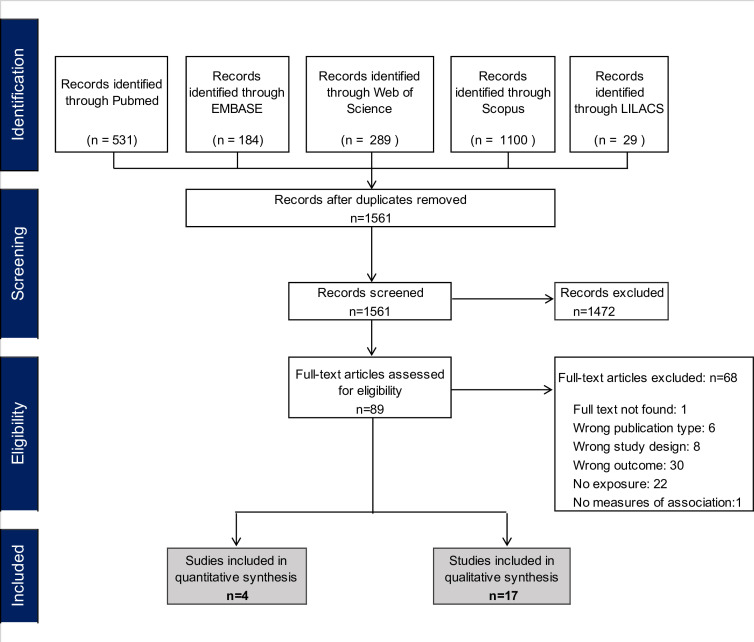
Preferred Reporting Items for Systematic Reviews and Meta-Analyses flowchart of included studies.

The Pubmed, Embase, Web of Science, LILACS and Scopus searches yielded 2133 articles, of which 572 were duplicates. Full texts were assessed of 89 potentially eligible studies. The main reasons for exclusion are listed in figure 1.

After exclusions, 4 quantitative papers, 1 mixed-methods paper and 16 qualitative papers were included. Two quantitative studies evaluated the association of D&A with PNC use,^50 51^ one with breast feeding^52^ and one with maternal PPD.^53^ All included qualitative studies evaluated D&A in relation to access to PNC.^15 54–69^ Of all included studies, 16 were conducted in Africa, 2 in Latin America (Brazil) and 2 in Asia (China and Indonesia). A summary of the studies is presented in tables 1 and 2.

**Table 1:** Characteristics of included quantitative studies

Study	Country	Study aims	Participants’ characteristics	Sample size	Study design	Exposure definition	Exposure prevalence	Outcome measured	Outcome prevalence
Bishanga *et al*^50^	Tanzania	To explore women’s experience of facility-based childbirth care, including D&A, choice of birth position, offer of a birth companion and perceived facility cleanliness.	Women aged 15–49 years who had given birth in health facilities during the 2 years preceding the survey.	732	Cross-sectional	Self-report of any of the following: left alone for a long period of time, left to deliver unassisted/alone, verbally abused, shared a bed with another person during labour, level of privacy, provided with no bed sheet, physical violence, inappropriate touching, discrimination, denied services, detained for payment, denied food/drink or care without consent.	73.1%	PNC use—any healthcare services given to women or baby by a professional health worker at a health facility within 48 hours of delivery.	Early postnatal check for women: 339 (46.3%); early postnatal check for baby: 358 (51.4%).
Creanga *et al*^51^	Malawi	To examine predictors of perinatal health service utilisation and to assess patient satisfaction with these services when last obtained.	Women aged 15–49 years who have given birth within the last 12 months and whose babies were alive at the time of the survey.	1301	Cross-sectional (baseline data from a cluster RCT)	Perceptions regarding the cleanliness of the facility, patients’ privacy, providers availability at the facility, quality of services offered, unmarried woman lack of access to services. Assessed by a 5-point agreement Likert scale.	Cleanliness: 3.5%; privacy: 6.7%; provider availability: 10.2%; low quality services: 10.9%; access to FP/RHs for unmarried women: 31.5%.	Maternal and neonatal PNC use—use after last delivery and number of checks within 2 months post partum.	77.5%
Bandeira de Sá *et al*^52^	Brazil	To identify factors associated with breast feeding in the first hour of life.	Mother–child pairs aged 0–12 months who attended health units.	1027	Cross-sectional	Self-report of any of the following during labour or delivery: physical violence (painful medical examination, being hit pushed or tied up), verbal violence (being yelled at), neglect (denial of care, fail to provide pain relief or lack on information about procedures), rooming in.	Verbal violence: 17.8%; physical violence: 17.3%; neglect: 16.7%; no rooming-in: 10.1%.	Child placed in the chest to breast feed in the first hour of life.	77.3%
Silveira *et al*^53^	Brazil	To examine the effect of the different types of disrespectful and abusive experiences on maternal postpartum depression occurrence and to explore if the associations differ according to women’s antenatal depressive symptoms status.	All women resident in the urban area, with confirmed pregnancy estimated delivery date in the year 2015.	3065	Cohort	Self-reported information on disrespect and abuse as any of the following: verbal abuse, denial of care (abandonment of care), physical abuse and undesired procedures (non-consented care) during the process of childbirth.	18.0% (95% CI 16.7 to 19.4)	Maternal postpartum depression- assessed by EPDS with cut-off of ≥13 points for moderate signs of depression and ≥15 points for severe signs of depression.	EPDS score ≥13: 9.4%; EPDS score ≥15: 5.7%

EPDS, Edinburgh Postnatal Depression Scale; FP, family planning services; PNC, postnatal care; RCT, Randomised controlled trial; RH, reproductive health services.

**Table 2:** Characteristics of included qualitative studies

Study	Country	Study aims	Participants’ characteristics	Study design and data collection	Aspects of D&A explored*
Chen *et al*^54^	China	To explore coverage, quality of care, reasons for not receiving care and barriers to providing postnatal care after introduction of new policy.	Caregivers of children younger than 2 years of age and township maternal and child healthcare workers.	Mixed methods combining a quantitative household survey and qualitative semi-structured interviews.	Health system level issues such as workload, income and training.
Dol *et al*^62^	Tanzania	To explore the experience of newborn care discharge education at a national hospital in Dar es Salaam, Tanzania from the perspective of mothers and nurse midwives.	Mothers who recently gave birth at national hospital and nurse midwives working on the postnatal and labour ward.	Qualitative descriptive research using in-depth interviews.	Woman-provider communication, and social, institutional and cultural influences when providing care.
Ganle and Dery^63^	Ghana	To explore the barriers to and opportunities for men’s involvement in maternal healthcare in the Upper West Region of Ghana.	Men and their spouses, community chiefs, women leaders, assembly men, community health nurses, community health officers and mother-to-mother support group leaders.	Qualitative focus group discussions, in-depth interviews and key informant interviews.	Challenges to male involvement in maternal healthcare, including institutional constraints and providers attitudes.
Kane *et al*^64^	Sudan	To gain insight into what hinders women from using maternal health services.	Community members, traditional leaders and traditional birth attendants.	Qualitative focus group discussions and in-depth interviews.	Social fears, social expectations and social interactions.
Mahiti *et al*^65^	Tanzania	To explore women’s views about the maternal health services (pregnancy, delivery and postpartum period) that they received at health facilities in rural Tanzania.	Women attending a health facility for vaccination at Kongwa District Hospital and Ugogoni Health Centre.	Qualitative focus group discussions and non-participant observation.	Women-provider interaction, waiting times, informal payments and material constraints (drug shortage and dirtiness).
McMahon *et al*^66^	Tanzania	To explore how rural Tanzanian women and their male partners describe disrespect and abuse experienced during childbirth in facilities and how they respond to abuse in the short or long-term.	Women, male partners, community health workers (CHWs) and community leaders from eight health centres across four districts.	Qualitative, cross-sectional study using in-depth interviews.	Types of verbal and physical abuse, discriminatory treatment, unpredictable financial charges and fear of detention.
Melberg *et al*^67^	Burkina Faso	To explore how communities in rural Burkina Faso perceive the promotion and delivery of facility pregnancy and birth care, and how this promotion influences health-seeking behaviour.	Women with recent health centre birth, women with a recent home birth, their partners and community men and women.	In-depth interviews and focus group discussions.	Fear of reprimands, economic sanctions, denial of care, stigma and discriminatory practices.
Mselle *et al*^59^	Tanzania	To examine how postpartum care was delivered in three postnatal healthcare clinics in Dar es Salaam, Tanzania.	Nurse-midwives and obstetricians from Dar es Salaam Referral Regional Hospitals.	Semi-structured interviews.	Relations of power among providers and women, focusing on beliefs, values, practices, language, meaning.
Morgan *et al*^68^	Uganda	To understand the role of gender power relations in relation to access to resources, division of labour, social norms and decision-making affect maternal healthcare access and utilisation in Uganda.	Women who had given birth recently, fathers whose wives had given birth recently, and transport drivers.	Qualitative focus group discussions.	Access to resources, division of labour (including male involvement), and social norms (including health workers attitudes and behaviours).
Ochieng and Odhiambo^69^	Kenya	To understand what factors are leading to low healthcare seeking during pregnancy, childbirth and postnatal period in Siaya County in Kenya.	Women attending ANC in Kenyan public primary healthcare facilities.	Qualitative focus group discussions.	Transportation issues, affordability, attitudes of health providers, embarrassment, autonomy in decision making, denial of care or punishment for delaying care.
Ongolly and Bukachi^55^	Kenya	To explore the barriers to men’s involvement in antenatal and postnatal care in Butula subcounty, Western Kenya.	Married men of the Butula subcounty who had had children in the past 1 year and healthcare workers in charge of maternal health services.	Mixed methods using quantitative surveys, focus group discussions and key informant interviews.	Health systems barriers including long waiting limes, lack of privacy, infrastructure constraints and providers’ attitudes.
Probandari *et al*^56^	Indonesia	To explore barriers to utilisation of postnatal care at the village level in Klaten district, Central Java Province, Indonesia.	Mothers with postnatal complications, family members and village midwives.	Qualitative data using in-depth interviews.	Suboptimal patient-centred care including lack of communication, availability of providers, insufficient time, inadequate education, selective care, cultural beliefs and practices, social power.
Sialubanje *et al*^60^	Zambia	To identify psychosocial and environmental factors contributing to low utilisation of maternal healthcare services in Kalomo, Zambia.	Women of reproductive age (15–45 years) who gave birth within the last year, traditional leaders, mothers, fathers, community health workers and nurse-midwives.	Qualitative focus group discussions and in-depth interviews.	Provider’s attitude such as verbal abuse and health systems constraints.
Sacks *et al*^15^	Uganda and Zambia	To examine experiences with, and barriers to, accessing postnatal care services in the context of a maternal health initiative.	Women who had delivered in the preceding year and lived within the eight districts.	Qualitative focus group discussions.	Fear of verbal or physical abuse, fear of denial of care or threat of denial of care, and neglect.
Yakong *et al*^57^	Ghana	To describe rural women’s perspectives on their experiences in seeking reproductive care from professional nurses.	Women 15 and 49 years of age and who had received care from two rural clinics and clinic nurses and community-based surveillance volunteers.	Qualitative study with in-depth interviews, focus group discussions and participant observation.	Intimidation and verbal abuse, experiences of limited choices, of receiving silent treatment and of lack of privacy.
Yevoo *et al*^58^	Ghana	To explore ‘how’ and ‘why’ pregnant women in Ghana control their past obstetric and reproductive information as they interact with providers at their first antenatal visit, and how this influences providers’ decision-making at the time and in subsequent care encounters.	Pregnant women who were within a gestational age of between 12 and 20 weeks and focus group discussions with pregnant and postnatal women.	Ethnographic study using participant observation, semi-structured interviews, and focus group discussions.	Healthcare providers’ ideological ‘domination and humiliation, including derogatory comments and verbal abuse, stigmatisation and discrimination, privacy and confidentiality.
Zamawe *et al*^61^	Malawi	To examine the perceptions of parents toward the postpartum period and postnatal care in order to deepen the understanding of the maternal care-seeking practices after childbirth.	Women and men who had either given birth or fathered a baby within 12 months prior to the study (new parents).	Descriptive qualitative study using focus group discussions.	Health system constraints related to long waiting times, costs, distance.

*The information presented in this column has been extracted during the initial coding phase of the qualitative analysis. No explicite conceptual definition of D&A was provided in most of the included studies.

D&A, disrespect and abuse.

### Quantitative synthesis of main outcomes

**Figure 2:**
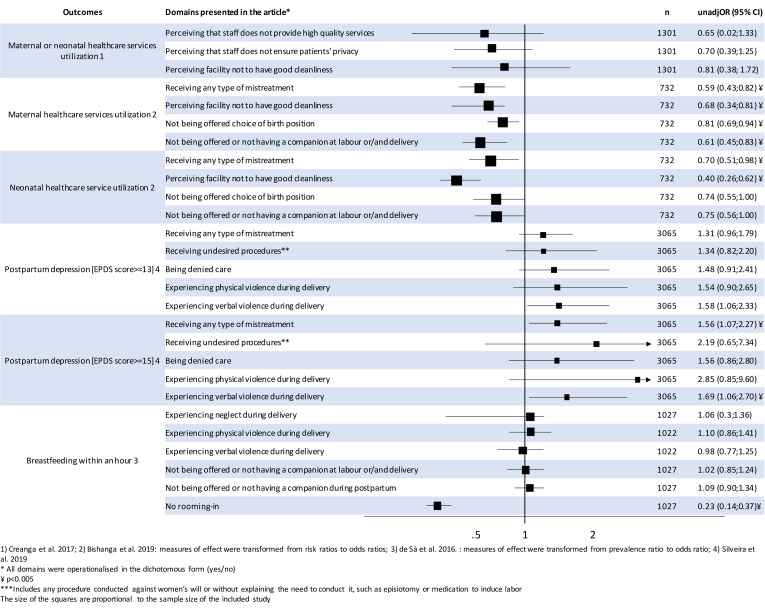
Summary of quantitative findings of the association between different domains of disrespect and abuse and PNC utilisation, breastfeeding and postpartum depression. (1) Creanga *et al*;^51^ (2) Bishanga *et al*:^50^ measures of effect were transformed from risk ratios to ORs; (3) Bandeira de Sà *et al*:^52^ measures of effect were transformed from prevalence ratio to OR; (4) Silveira *et al*.^53^ *All domains were operationalised in the dichotomous form (yes/no). ¥p<0.005. ***Includes any procedure conducted against women’s will or without explaining the need to conduct it, such as episiotomy or medication to induce labour. EPDS, Edinburgh Postnatal Depression Scale.

All quantitative studies defined D&A and the outcomes differently. Table 3 shows how the D&A domains extracted from the included studies relate to existing typologies of D&A and MDC. In this section, we present a narrative summary of the findings further illustrated in figure 2.

**Table 3:** Categorisation of the domains of D&A extracted from included quantitative studies based on existing typologies

Domains as extracted from article	Domains categorised based on D&A typology*	Domains categorised based on MDC typology†
Experiencing physical violence during delivery‡§	Physical abuse	Physical abuse
Experiencing verbal violence during delivery§	Non-dignified care	Verbal abuse
Receiving undesired procedures§		Failure to meet professional standards of care
Being denied care§		
Experiencing neglect during delivery‡		
Perceiving that staff does not provide high quality services¶		
Not being offered choice of birth position**		Poor Rapport between women and providers
Not being offered or not having a companion at labour or/and delivery**‡	Abandonment of care	
Not being offered or not having a companion during post partum‡		
No rooming-in‡		
Perceiving that staff does not ensure patients’ privacy¶	Non-confidential care	Health systems conditions and constraints
Perceiving facility not to have good cleanliness¶**	Non-apply	
Receiving any type of mistreatment**§	Receiving any type of D&A **§	Receiving any type of mistreatment**§

*Source: Browser and Hill.^3^

†Source: Bohren *et al*.^4^

‡Bandeira de Sà *et al*.^52^

§Silveira *et al*.^53^

¶Creanga *et al.*^51^

**Bishanga *et al*.^50^

D&A, disrespect and abuse; MDC, mistreatment during childbirth.

In the study by Bishanga *et al*,^50^ the 73.1% of women who reported experiencing at least one form of D&A had 41% lower odds of receiving an early postnatal check (unadjusted OR: 0.59, 95% CI 0.43 to 0.82) and 30% lower odds of their newborn receiving an early postnatal check (95% CI 0.51 to 0.98) compared with mothers who did not experience D&A. The study by Silveira *et al*^53^ reported that women who experienced any D&A during childbirth (18%) had 56% higher odds (unadjusted OR: 1.56; 95% CI 1.07 to 2.27) of developing severe PPD compared with those that did not experience D&A. When analysed by domain of D&A, Silveira *et al* showed that if the abuse was verbal, women had 69% greater odds (unadjusted OR: 1.69; 95% CI 1.06 to 2.70) of developing severe PPD, compared with those that did not experience verbal abuse. Bandeira de Sà *et al*^52^ found that keeping the baby in the same room as the mother after delivery was the only clinically or statistically significant predictor of breast feeding within 1 hour (unadjusted OR: 0.23; 95% CI 0.14 to 0.37) among those measured, however, the survey was conducted with mothers attending vaccination centres, and so the study population may have already self-selected as those with high levels of engagement. Additionally, Bishanga *et al*^50^ reported that women not offered a choice of birth position had 19% lower odds of a postnatal check (95% CI 0.69 to 0.94); and those who perceived the facility as not being clean had 32% (OR=0.68; 95% CI 0.34 to 0.81) and 60% lower odds (95% CI 0.26 to 0.62) of receiving a maternal early postnatal check and newborn early postnatal check, respectively.

Creanga *et al*^51^ found no statistical association at the 5% level between postnatal check and any of the domains measured. The authors stated that this may be explained by a widespread perception of poor quality of care by women participating in the study.

### Qualitative synthesis of factors affecting PNC

The main objective of the qualitative analysis was to better understand if and how D&A and its underlying drivers affect the use of PNC. All included studies with a qualitative component described this relationship from different perspectives, however, no study had this as its primary research question. Six studies aimed to explore barriers to maternal and newborn health (MNH) care,^15 54 56 60 64 69^ five explored experience of MNH care,^57 59 62 65 66^ three evaluated perception of MNH care,^58 61 67^ two described male involvement in maternal and newborn care^55 63^ and one explored gender dynamics in care provision.^68^ The majority of the studies (15/17; 88%) were conducted in Africa (Burkina Faso,^67^ Ghana,^57 58 63^ Malawi,^61^ Kenya,^55 69^ Uganda,^15 68^ Sudan,^64^ Zambia^15^ and Tanzania^59 62 65 66^) while the remaining two were from Asia (China and Indonesia).^54 56^ While women direct experience of D&A during childbirth was identified as a factor influencing their decision to accessing care, two other themes emerged from the included studies to better explain the underlying factors driving such relationship. The assessment of confidence in the review findings showed high confidence in the theme related to women’s direct experience and low to moderate confidence in the remaining two themes (online supplemental appendix 4).

### Theme 1: women’s direct experience

The first theme that appeared repeatedly was the effect of ‘women’s direct experience’, indicating that a previous negative interaction with a health provider could impact women’s subsequent care-seeking behaviour either by changing provider or by delaying or avoiding care altogether. This theme includes aspects related to health systems constraints and prior experiences of mistreatment.

#### Health systems constraints

Inadequate infrastructure and staff shortage contributed to the loss of trust in the maternal and neonatal services women received.^54 59–61 63 65^ Women frequently reported having to wait before receiving care, which resulted in a poor patient/client relation.^61^ Although in some of the included articles, women accepted the long waiting times as the result of a limited number of staff, in others they questioned the value of PNC as other issues were prioritised before theirs.^54 55 61^ Men and women used the long waiting time as an argument for lack of male involvement in MNH care, as men were frequently the ones in the paid workforce and often perceived themselves not to be ‘in a position to spend the day waiting for their wives to receive care’.^63 65^ Men also reported a shortage of waiting space for them as a reason for not participating in MNH care, often being asked to wait outside while women are were treated.

Women referred to facility cleanliness as another major deterrent for accessing PNC.^59 65^ They described labour wards as dirty and untidy, sometimes having to reuse dirty bed sheets or share a bed with other women, all of which impacts strongly on their confidence in the hygiene of the health facilities.

#### D&A during previous contacts with health system

Many women referred to their previous experience with the healthcare system as a barrier to PNC use.^15 56–60 62 68^ Women identified areas where they felt that nurses did not provide them with sufficient, clear or timely information about the postnatal period, including skin-to-skin contact, hygiene practices and positioning for breast feeding.^62^ In one article, they mentioned that education on postnatal practices was provided immediately after delivery when women were still in pain.^56^ Nurses-midwives also recognised their lack of time for providing health education to new mothers or even providing essential life-saving practices to mothers/newborns because of staff shortages.^59^

Another recurring theme that women mentioned as having profound effect on their health-seeking patterns was the lack of privacy during the visits.^57 58^ In some articles, women expressed concerns about sharing confidential information because they felt that other people could listen into their interactions with health providers and questioned healthcare providers’ ability to protect the confidentiality of the information they exchanged. Women recognised that these issues prevented them from discussing topics related to their reproductive health, contraceptive use or ill-health as they were afraid of negative repercussions on their relationship with family members or their husbands.

Many women and men identified rudeness and abusive behaviours by health workers as a key problem affecting access to and use of maternal and neonatal services.^15 58 68^ Women described nurses as ‘rude and harsh’, with many complaining about receiving ‘verbal abuse’, ‘condescension’ or ‘derogatory comments’.^68^ As described in a group discussion with young mothers: ‘the nurses beat you when you refuse to push’.^58^

### Theme 2: women’s expectations

The second theme was ‘women’s expectations’, meaning the apprehension of attending facilities based on the fear of what is expected of them by the healthcare provider. As an example, this includes women’s sense that they could be shamed for the ill-health of their child or for not following the recommendations of health providers.

#### Internalised stigma

Women’s internalised stigma frequently appears as a deterrent to PNC use in the form of fear of repercussions and embarrassment.^15 64 66–69^ In some studies, women describe their fear of being detained at the health facility or ‘shamed and belittled’ for not having enough money to pay for services.^68^ Others were afraid of reprimand and embarrassment from health workers because they lacked proper baby clothing, and they believed that appearing dishevelled and uncared for gave an impression of not being celebrated and dignified by the family.^64 66^ Women also reported that if they failed to honour what the health provider expected of them, they would be made to wait, yelled at or criticised.^64^ However, these fears were most prominent among women who delivered at home.^15^

Women avoided accessing PNC in all these cases instead of confronting the health providers as they were afraid that they would be denied future care or services. Women described that they did not consider themselves competent enough to engage in open confrontation fearing they would have to seek care in another facility further away from their place of residence, which would impact on the cost and time to access healthcare when needed.^69^

#### Beliefs and traditions

Some women discussed differences between medical and traditional knowledge as major barriers for accessing the postnatal clinic.^56 62 69^ The lack of culturally sensitive care during childbirth, the ineffective communication, and the dismissal of traditional practices and beliefs led to women avoiding access to subsequent care. Women reported unwanted medical interventions as a reason for not attending PNC, with vaccine hesitancy due to the fear that an injection could harm the child as one of the main concerns for avoiding PNC.

### Theme 3: women’s agency

The last analytical theme was ‘women’s agency’ referring to larger societal or familial influences that diminishe women’s decision-making power, a consequence of health systems failures and ineffective education opportunities after childbirth.

#### Male involvement and gender dynamics

The lack of participation of men in maternity care was described in many studies as a healthcare system’s failure to actively engage men on issues of maternal health, with many men reporting negative attitudes from health workers when trying to get involved in the childbirth experience.^55 59 63 68^ While some healthcare workers agreed that family members needed to be included in post-delivery education, they often mentioned restrictions on this practice due to space constraints or other infrastructural issues.^59^ This, in conjunction with traditional gender norms and cultural beliefs, led to most men perceiving maternal and newborn care as a ‘feminine’ domain, disengaging themselves from the process of care.^59 63 68^

Both men and women acknowledged that, even if healthcare is perceived as the responsibility of the woman, men still exercised their power by either permitting or restricting women’s access to services, through financial control or other forms of domestic violence.^55 63 68^ Thus, women avoided PNC as any delay that prevented them from performing their household chores or accepting care practices condemned by their partner, could potentially trigger episodes of domestic violence.

#### Family and societal influence (social norms)

The suboptimal provision of education on PNC after facility childbirth made women less prepared to confront external family and societal influences after discharged.^56 59 62 69^ Midwives reported not having adequate time to build a trusting relationship with women to discuss issues related to postpartum care because of staff shortages or space constraints, while women claimed they did not understand midwives’ instructions on how to care for the baby as they rushed through it and used high-manner language.^62^

The lack of preparation for the postnatal period meant that many women, especially those who share homes with their extended family (such as their in-laws or their grandparents), were more likely to follow culture-related myths and rules passed by their relatives. 56 59 69 Women recognised they were expected to obey traditional family rules rather than acting on any teaching provided at the hospital.^56^ Thus, they would refrain from accessing PNC due to fear of repercussions for not following providers’ instructions during previous contact.

Although this theme appeared less frequently across articles, health providers recognised the cultural beliefs and traditional practices regarding healthcare in their communities and mentioned that they try to discuss this issue with women. However, they acknowledged that family and society’s influences are particularly strong during the postnatal stage.

## Discussion

This systematic review aimed to understand how and why the experience that women and newborns have during childbirth can impact on their relationship with the healthcare system and on their health and well-being. Different domains of D&A were associated with poorer engagement with early maternal care, early neonatal care and PPD; the only domain associated with breast feeding was rooming-in, as mothers and babies are kept together promoting opportunities of contact. Although there is currently a paucity of high-quality quantitative evidence and lack of consistency in the measurement of the exposure, the themes that emerged from the qualitative studies could indicate different pathways by which these associations could hold true. These pathways reflect multiple interrelated influences that guide women to access and use PNC, and subsequently impact on their health and that of their newborn.

Echoing our quantitative results, the qualitative findings suggest that the quality of medical care received by women directly influences women’s healthcare seeking behaviours. As evidence shows that a negative experience during antepartum care is a barrier to facility-based childbirth,^5^ a negative experience during facility-based childbirth can also influence the decision to seek care postnatally. Despite interpersonal factors being the most prominent contributors to a negative experience of care across the identified literature, system level conditions also play a crucial role. Health system constraints, such as staff shortages and lack of cleanliness, often associated with longer waiting times and poorer quality of care, can create an environment in which women feel unwelcome and discouraged to return for future visits. However, the disrespectful or abusive treatment received by women, including health system constrains, appear not to be sufficient to solely explain the potential impact on PNC use.

Our findings show that women’s decision-making process on PNC seeking originates from a complex intersection of factors, both from within and outside the healthcare realm. It is influenced by broader cultural, social and gender norms that reify women’s vulnerabilities within society, not only as part of their direct experience with healthcare. The most disenfranchised women are more likely to avoid institutional healthcare as another place where they might feel disempowered, a consequence of their ‘internalised stigma’, and systemic disadvantages. This aligns with Dixon-Woods concept of ‘candidacy’ to describe inequity of access to health services and health outcomes.^70^ *Candidacy* suggests that an individual’s identification of his or her ‘legitimacy’ for health services is structurally, culturally, organisationally and professionally construed; with a range of characteristics, such as gender, poverty, education, age and ethnicity coalescing to suppress the use of services.^71^ This combination of systemic disadvantages can reduce women’s agency, diminish her candidacy and compromise her access to healthcare.^72^ This might partially explain why, even in settings with universal healthcare provision, those in deprived circumstances make less use of services than the more affluent.

Our findings constitute the initial necessary steps to bring clarity on D&A as a possible barrier to care and to women and newborn’s health and well-being. This review highlighted several gaps of knowledge in the current literature. The most prominent one comes from the methodological challenges in quantifying and comparing the prevalence of D&A and its impacts across studies and settings, as no unique definition was used. In recent years, efforts have been made to develop universal evidence-based definitions, typologies and measurement tools.^73–75^ The widespread adoption of these tools could allow for a better harmonisation of measures in future studies. Moving forward, we need to be strategic in addressing the difficulties attached to such a complex phenomenon. More research is needed to develop and evaluate interventions to tackle the structural drivers sustaining D&A, such as damaging gender norms, social inequalities and asymmetric power distributions that promote the normalisation of poor treatment. Alongside this, we need measurable objectives that are attainable in the short term and help move us towards those broader systemic changes. Understanding the immediate health benefit of providing respectful maternity and newborn care can be a first strategic step to encourage health workers to equate the value of non-clinical aspects of care to that of high-quality, evidence-based clinical practices. In this review, we selected specific public health outcomes that can bring a new perspective to tackling this issue and can contribute to designing custom-made messages to address front-line stakeholders. We highlight the need for primary research to robustly measure the health and well-being impact of D&A in order to quantify and monitor progress as interventions are put in place.

### Limitations and strength of the review

While some of the cross-sectional studies show preliminary evidence of a possible relation between D&A during childbirth with PNC utilisation and maternal mental health, the results come from small scale studies with a low prevalence of the exposure and provide inconclusive evidence. The low prevalence could be explained by recall or social desirability biases as studies required women to remember what happened during childbirth or were conducted within hospital settings. Additionally, the confidence in the qualitative evidence related to broader cultural and societal themes was low to moderate, highlighting the need to further study how structural aspects interplay with D&A and PNC. Further, the definitions of D&A and outcomes differed between studies making cross-study comparisons challenging. Thus, the potential for the complementarity of quantitative and qualitative methods for synthesising data could not be fully exploited. Finally, the limited number of studies from Asia and Latin America relative to Africa could be affecting generalisability.

There are several strengths to this review as it is, to our knowledge, the first to summarise the consequences of D&A during childbirth. The use of mixed methods allows for a more comprehensive understanding of the available evidence, integrating the measurement of the effect size of the association with the identification of broader factors that interact to bring about such effect. Following a systematic process for the screening, inclusion and analysis of the retrieved articles, this review shows reliable and transparent results that highlight the need for further research in this field.

## Conclusion

Women’s access to PNC can be influenced by a myriad of factors with long lasting effects on her health and her newborn’s. In the quest to improve women and newborns’ health and guarantee access to high-quality, respectful, dignified and supportive care, understanding the consequences of a negative birth experience can provide a step forward in prioritising the problem. While a complex, systemic and multidimensional response is needed, it might take longer to materialise and will require buy-in from multiple stakeholders. This review aims to offers a new perspective to the issue of D&A and calls on the public health community to urgently address D&A during facility-based childbirth for the sake of its potentially damaging health consequences.

## Data Availability

Data are available upon request. Data analysed in the current study will be made available from the corresponding author on reasonable request.
