# Supplementary material for: Disrespect and abuse as a predictor of postnatal care utilisation and maternal-newborn well-being: a mixed-methods systematic review
Source: BMJ Glob Health. 2021 Apr 21;6(4):e004698. doi: 10.1136/bmjgh-2020-004698 (PMC8061800; doi:10.1136/bmjgh-2020-004698)
Supplement: Supplementary data [file bmjgh-2020-004698supp001.pdf]

Supplementary material

Table of Contents

Supplementary material 1
Appendix 1. Search Strategy (10th March 2020) 2
PUBMED 2
EMBASE 3
WEB OF SCIENCE 4
SCOPUS 4
LILACS 5
Appendix 2. Quality assessment of quantitative studies included in the review* 6
Appendix 3. Quality assessment of qualitative studies included in the review* 7
Appendix 4. Assessment of confidence of qualitative findings: CERQual approach 8
Appendix 5. Summary of quantitative findings of the association between different domains of disrespect and abuse during childbirth and outcomes as reported in original article 10

Minckas N, *et al. BMJ Global Health* 2021; 6:e004698. doi: 10.1136/bmjgh-2020-004698

Appendix 1. Search Strategy (10th March 2020)

PUBMED

Mistreatment	#1	"disrespect"[tw] OR "disrespects"[tw] OR "disrespectful"[tw] OR "disrespected"[tw] OR "respectful"[tw] OR "abuse"[tw] OR "abused"[tw] OR "abusive"[tw] OR "abuses"[tw] OR "neglect"[tw] OR "neglected"[tw] OR "neglects"[tw] OR "confidentiality"[tw] OR "confidential"[tw] OR "non-confidential"[tw] OR "informed consent"[tw] OR "violence"[tw] OR "violent"[tw] OR "humiliation"[tw] OR "humiliate"[tw] OR "condescend"[tw] OR "condescending"[tw] OR "condescension"[tw] OR "intimidation"[tw] OR "intimidate"[tw] OR "yelling"[tw] OR "yell"[tw] OR "non dignified"[tw] OR "non-dignified"[tw] OR "undignified"[tw] OR "discrimination"[tw] OR "discriminate"[tw] OR "abandon"[tw] OR "abandonment"[tw] OR "detention"[tw] OR "human rights"[tw] OR "maltreatment"[tw] OR "mistreatment"[tw] OR "humanization"[tw] OR "humanized"[tw] OR "dehumanized"[tw] OR "dehumanization"[tw] OR "dignified"[tw] OR "undignified"[tw] OR "stigma"[tw] OR "dignity"[tw] OR "bullying"[tw] OR "bully"[tw] OR "Patient-Centered Care" [Mesh]	**671720**
	#2	"confidentiality"[mesh] OR "informed consent"[mesh] OR "women's rights"[mesh] OR "violence"[mesh] OR "social stigma"[mesh] OR "health services/ethics"[mesh] OR "health care quality, access, and evaluation/ethics"[mesh]	**212172**
	#3	#1 OR #2	**720410**
Facility based deliveries	#4	"facility based delivery"[tw] OR "facility based deliveries"[tw] OR "facility delivery"[tw] OR "facility deliveries"[tw] OR "facility based births"[tw] OR "facility based birth"[tw] OR "facility-based childbirth"[tw] OR "facility-based child birth"[tw] OR "facility birth"[tw] OR "facility births"[tw] OR "clinic delivery"[tw] OR "clinic deliveries"[tw] OR "clinic births"[tw] OR "clinic birth"[tw] OR "hospital delivery"[tw] OR "hospital deliveries"[tw] OR "hospital birth"[tw] OR "hospital births"[tw] OR "hospital childbirth"[tw] OR "hospital childbirths"[tw] OR "hospital based deliveries"[tw] OR "hospital based delivery"[tw] OR "hospital based births"[tw] OR "institutional birth"[tw] OR "institutional births"[tw] OR "institutional childbirth"[tw] OR "institutional childbirths"[tw] OR "institutional delivery"[tw] OR "institutional deliveries"[tw]	**3125**
	#5	"perinatal service"[tiab] OR "peri natal service"[tiab] OR "perinatal services"[tiab] OR "peri natal services"[tiab] OR "perinatal health service"[tiab] OR "peri natal health service"[tiab] OR "perinatal health services"[tiab] OR "peri natal health services"[tiab] OR "maternal care"[tiab] OR "maternal health care"[tiab] OR "maternal healthcare"[tiab] OR "maternal service"[tiab] OR "maternal health service"[tiab] OR "maternal services"[tiab] OR "maternal health services"[tiab]	**5652**
	#6	(#4 OR #5) AND #3	**756**
PNC	#7	"Postnatal Care"[Mesh] OR "Maternal-Child Health Services"[Mesh] OR"post natal service" [tiab] OR "postnatal service*"[tiab] OR "postnatal health service"[tiab] OR "post natal health service"[tiab] OR "postnatal health services"[tiab] OR "postnatal care"[tiab] OR "neonatal care"[tiab] OR "Postpartum Period"[Mesh] OR "postpartum care"[tw] OR "post partum care"[tw]	**74478**
Outcomes	#8	****MATERNAL MENTAL HEALTH AND WELLBEING:** "Depression, Postpartum"[Mesh] OR "Puerperal Disorders"[Mesh] OR "Depressive Disorder"[Mesh] OR "Depression" [tw] OR "postpartum depression" [tw] OR "maternal depression"[tiab] OR "perinatal depression" [tiab] OR "mental disorder" OR "adjustment disorder" OR "affective disorder" OR "dysthymic disorder" OR "psychiat*" OR "behaviour control" OR "psychological phenomena" OR "depression" OR "mental health" OR "stress disorder" OR "anxiety disorder" OR "maternal welfare" OR "maternal health"	**719092**
	#9	**** FEEDING PRACTICES** "Breast Feeding"[Mesh] OR "Feeding Behavior"[Mesh] OR breastfeed* [tiab] OR "child nutrition" OR "Lactation"[Mesh]	**218485**
	#10	**** INFANT HEALTH** "Infant weight gain" [tiab] OR "newborn weight gain" [tiab] OR "wasting" [tiab] OR ("Weight gain" [Mesh] AND "Infant" [Mesh]) OR "Infant, low birth weight" [Mesh]	**54007**
Final	#11	**#6 AND (#7 OR #8 OR #9 OR #10)**	**531**

Minckas N, *et al. BMJ Global Health* 2021; 6:e004698. doi: 10.1136/bmjgh-2020-004698

EMBASE

	Searches	Results
1	(disrespect* or respectful or abuse* or neglect* or confidential* or non-confidential* or humiliat* or violence or violent* or condescen* or yell* or abandon* or dignified or non-dignified or discriminat* or maltreatment* or mistreatment or humaniz* or dehumaniz* or undignified or dignity or stigma or bully*).tw.	729421
2	(perinatal service* or peri natal service* or perinatal health service* or peri natal health service* or maternal care or maternal health care or maternal healthcare or maternal service* or maternal health service*).ab.	4469
3	(facility based deliver* or facility deliver* or facility based birth* or facility birth* or facility-based child birth* or facility-based childbirth* or clinic deliver* or hospital birth* or hospital childbirth* or hospital based deliver* or hospital based birth* or institutional birth* or institutional childbirth* or institutional deliver*).tw.	3035
4	(postnatal care* or maternal-child health services* or post natal service* or postnatal service* or postnatal health service* or post natal health service* or neonatal care* or postpartum period* or postpartum care* or post partum care*).tw.	18380
5	(depressive disorder* or depression* or postpartum depression* or maternal depression* or perinatal depression or mental disorder* or adjustment disorder* or affective disorder* or dysthymic disorder* or psychiat* or behaviour control or psychological phenomena or mental health or stress disorder* or anxiety disorder* or maternal welfare or anxiety).tw.	949147
6	(mother-child relation* or attachment* or bonding or child attachment or newborn attachment or maternal-newborn attachment).tw.	184933
7	(breast feeding or feeding behavior or breastfeed* or child nutrition or lactation).tw.	83600
8	(infant weight gain or newborn weight gain or wasting or (weight gain and infant*)).tw.	29597
9	2 or 3	7176
10	1 and 9	627
11	4 and 10	38
12	5 or 6 or 7 or 8	1233478
13	10 and 12	151
14	11 or 13	184

Minckas N, et al. BMJ Global Health 2021; 6:e004698. doi: 10.1136/bmjgh-2020-004698

WEB OF SCIENCE

Set	Results	Save History
# 14	289	#13 OR #11
# 13	262	#12 AND #10
# 12	2,406,740	#8 OR #7 OR #6 OR #5
# 11	33	#10 AND #4
# 10	711	#9 AND #1
# 9	6,345	#3 OR #2
# 8	363,541	TS=("infant weight gain" OR "newborn weight gain" OR wasting OR ("weight gain" AND infant*))
# 7	100,461	TS=("breast feeding" OR "feeding behavior" OR "feeding behaviour" OR breastfeed* OR "child nutrition" OR lactation)
# 6	1,049,938	TS= ("mother-child relation" OR attachment* OR bonding OR "child attachment" OR "newborn attachment" OR "maternal-newborn attachment")
# 5	919,639	TS=("depressive disorder" OR depression* OR "postpartum depression" OR "maternal depression" OR "perinatal depression" OR "mental disorder" OR "adjustment disorder" OR "affective disorder" OR "dysthymic disorder" OR psychiat* OR "behaviour control" OR "psychological phenomena" OR "mental health" OR "stress disorder" OR "anxiety disorder" OR "maternal welfare" OR "anxiety")
# 4	12,112	TS=("postnatal care" OR "maternal-child health services" OR "post natal service" OR "postnatal service" OR "postnatal health service" OR "post natal health service" OR "neonatal care" OR "postpartum period" OR "postpartum care" OR "post partum care")
# 3	1,158	TS=("facility based deliver"OR "facility deliver" OR "facility based birth" OR "facility birth" OR "facility-based child birth" OR "facility-based childbirth" OR "clinic deliver" OR "hospital birth" OR "hospital childbirth" OR "hospital based delivery" OR "hospital based birth" OR "institutional birth" OR "institutional childbirth" OR "institutional delivery")
# 2	5,325	TS=("perinatal service" OR "peri natal service" OR "perinatal health service" OR "peri natal health service" OR "maternal care" OR "maternal health care" OR "maternal healthcare" OR "maternal service" OR "maternal health service")
# 1	1,034,335	TS=(disrespect* OR respectful OR abuse* OR neglect* OR confidential* OR non-confidential* OR humiliat* OR violence OR violent* OR condescen* OR yell* OR abandon* OR dignified OR non-dignified OR discriminat* OR maltreatment* OR mistreatment OR humaniz* OR dehumaniz* OR undignified OR dignity OR stigma OR bully*)

SCOPUS

(((TITLE-ABS-KEY (disrespect* OR respectful OR abuse* OR neglect* OR confidential* OR non-confidential* OR humiliat* OR violence OR violent* OR condescen* OR yell* OR abandon* OR dignified OR non-dignified OR discriminat* OR maltreatment* OR mistreatment OR humaniz* OR dehumaniz* OR undignified OR dignity OR stigma OR bully*)) AND ((TITLE-ABS-KEY ("perinatal service" OR "peri natal service" OR "perinatal health service" OR "peri natal health service" OR "maternal care" OR "maternal health care" OR "maternal healthcare" OR "maternal service" OR "maternal health service")) OR (TITLE-ABS-KEY ("facility based deliver" OR "facility deliver" OR "facility based birth" OR "facility birth" OR "facility-based child birth" OR "facility-based childbirth" OR "clinic deliver" OR "hospital birth" OR "hospital childbirth" OR "hospital based delivery" OR "hospital based birth" OR "institutional birth" OR "institutional childbirth" OR "institutional delivery")))) AND (TITLE-ABS-KEY ("postnatal care" OR "maternal-child health services" OR "post natal service" OR "postnatal service" OR "postnatal health service" OR "post natal health service" OR "neonatal care" OR "postpartum period" OR "postpartum care" OR "post partum care"))) OR (((TITLE-ABS-KEY (disrespect* OR respectful OR abuse* OR neglect* OR confidential* OR non-confidential* OR humiliat* OR violence OR violent* OR condescen* OR yell* OR abandon* OR dignified OR non-dignified OR discriminat* OR maltreatment* OR mistreatment OR humaniz* OR dehumaniz* OR undignified OR dignity OR stigma OR bully*)) AND ((TITLE-ABS-KEY ("perinatal service" OR "peri natal service" OR "perinatal health service" OR "peri natal health service" OR "maternal care" OR "maternal health care" OR "maternal healthcare" OR "maternal service" OR "maternal health service")) OR (TITLE-ABS-KEY ("facility based deliver" OR "facility deliver" OR "facility based birth" OR "facility birth" OR "facility-based child birth" OR "facility-based childbirth" OR "clinic deliver" OR "hospital birth" OR "hospital childbirth" OR "hospital based delivery" OR "hospital based birth" OR "institutional birth" OR "institutional childbirth" OR "institutional delivery")))) AND ((TITLE-ABS-KEY ("depressive disorder" OR depression* OR "postpartum depression" OR "maternal depression" OR "perinatal depression" OR "mental disorder" OR "adjustment disorder" OR "affective disorder" OR "dysthymic disorder" OR psychiat* OR "behaviour control" OR "psychological phenomena" OR "mental health" OR "stress disorder" OR "anxiety disorder" OR "maternal welfare" OR "anxiety")) OR (TITLE-ABS-KEY ("mother-child relation" OR attachment* OR bonding OR "child attachment" OR "newborn attachment" OR "maternal-newborn attachment")) OR (TITLE-ABS-KEY ("breast feeding" OR "feeding behavior" OR "feeding behaviour" OR breastfeed* OR "child nutrition" OR lactation)) OR (TITLE-ABS-KEY ("infant weight gain" OR "newborn weight gain" OR wasting OR ("weight gain" AND infant*)))))
N=1100

LILACS

(((disrespect* OR respectful OR abuse* OR neglect* OR confidential* OR non-confidential* OR humiliat* OR violence OR violent* OR condescen* OR yell* OR abandon* OR dignified OR non-dignified OR discriminat* OR maltreatment* OR mistreatment OR humaniz* OR dehumaniz* OR undignified OR dignity OR stigma OR bully*) AND (tw:(perinatal service OR peri natal service OR perinatal health service OR peri natal health service OR maternal care OR maternal health care OR maternal healthcare OR maternal service OR maternal health service OR obstetric*)) OR (tw: (facility based deliver OR facility deliver OR facility based birth OR facility birth OR facility-based child birth OR facility-based childbirth OR clinic deliver OR hospital birth OR hospital childbirth OR hospital based delivery OR hospital based birth OR institutional birth OR institutional childbirth OR institutional delivery))) AND (tw: (postnatal care OR maternal-child health services OR post natal service OR postnatal service OR postnatal health service OR post natal health service OR neonatal care OR postpartum period OR postpartum care OR post partum care))) OR (((tw: (disrespect* OR respectful OR abuse* OR neglect* OR confidential* OR non-confidential* OR humiliat* OR violence OR violent* OR condescen* OR yell* OR abandon* OR dignified OR non-dignified OR discriminat* OR maltreatment* OR mistreatment OR humaniz* OR dehumaniz* OR undignified OR dignity OR stigma OR bully*)) AND ((tw:(obstetric* OR perinatal service OR peri natal service OR perinatal health service OR peri natal health service OR maternal care OR maternal health care OR maternal healthcare OR maternal service OR maternal health service)) OR (tw: (facility based deliver OR facility deliver OR facility based birth OR facility birth OR facility-based child birth OR facility-based childbirth OR clinic deliver OR hospital birth OR hospital childbirth OR hospital based delivery OR hospital based birth OR institutional birth OR institutional childbirth OR institutional delivery)))) AND ((tw:(depressive disorder OR depression* OR postpartum depression OR maternal depression OR perinatal depression OR mental disorder OR adjustment disorder OR affective disorder OR dysthymic disorder OR psychiat* OR behaviour control OR psychological phenomena OR mental health OR stress disorder OR anxiety disorder OR maternal welfare OR anxiety)) OR (tw:(breast feeding OR feeding behavior OR feeding behaviour OR breastfeed* OR child nutrition OR lactation)) OR (tw: (infant weight gain OR newborn weight gain OR wasting OR (weight gain AND infant*)))))
LILACS: 29

Appendix 2. Quality assessment of quantitative studies included in the review

	Creanga 2017	Bishanga 2019	De Sá 2016	Silveira 2019
**Criteria**				
1. Was the research question or objective in this paper clearly stated?	YES	YES	YES	YES
2. Was the study population clearly specified and defined?	YES	YES	YES	YES
3. Was the participation rate of eligible persons at least 50%?	CD	YES	CD	YES
4. Were all the subjects selected or recruited from the same or similar populations (including the same time period)? Were inclusion and exclusion criteria for being in the study prespecified and applied uniformly to all participants?	YES	YES	YES	YES
5. Was a sample size justification, power description, or variance and effect estimates provided?	NO	YES	YES	NO
6. For the analyses in this paper, were the exposure(s) of interest measured prior to the outcome(s) being measured?	NO	NO	NO	YES
7. Was the timeframe sufficient so that one could reasonably expect to see an association between exposure and outcome if it existed?	NA	NA	NA	YES
8. For exposures that can vary in amount or level, did the study examine different levels of the exposure as related to the outcome (e.g., categories of exposure, or exposure measured as continuous variable)?	NO	NO	NO	YES
9. Were the exposure measures (independent variables) clearly defined, valid, reliable, and implemented consistently across all study participants?	YES	YES	YES	YES
10. Was the exposure(s) assessed more than once over time?	NA	NA	NA	NO
11. Were the outcome measures (dependent variables) clearly defined, valid, reliable, and implemented consistently across all study participants?	YES	YES	YES	YES
12. Were the outcome assessors blinded to the exposure status of participants?	NA	NA	NA	NO
13. Was loss to follow-up after baseline 20% or less?	NA	NA	NA	YES
14. Were key potential confounding variables measured and adjusted statistically for their impact on the relationship between exposure(s) and outcome(s)?	YES	YES	YES	YES

**Overall quality***	M	H	M	H

*Overall quality was defined as high" (≥75% of applicable criteria), "medium" (50-<75% of applicable criteria) or "low" (<50% of applicable criteria) quality
Tool: NIH Quality Assessment Tool for Observational Cohort and Cross-Sectional Studies.

Appendix 3. Quality assessment of qualitative studies included in the review*

	**Section A: Are the results valid?**						**Section B: What are the results**			**Section C: Will the results help locally?**	Quality
	1. Was there a clear statement of the aims of the research?	2. Is a qualitative methodology appropriate?	3. Was the research design appropriate to address the aims of the research?	4. Was the recruitment strategy appropriate to the aims of the research?	5. Was the data collected in a way that addressed the research issue?	6. Has the relationship between researcher and participants been adequately considered?	7. Have ethical issues been taken into consideration?	8. Was the data analysis sufficiently rigorous?	9. Is there a clear statement of findings?	10. How valuable is the research?	
Chen 2014	YES	YES	YES	YES	YES	NO	YES	YES	YES	+	H
Dol 2019	YES	YES	YES	YES	YES	YES	YES	CT	YES	+	H
Ganle 2015	YES	YES	YES	YES	YES	YES	YES	YES	YES	+	H
Kane 2018	YES	YES	YES	YES	YES	YES	YES	YES	YES	+	H
Mahiti 2015	YES	YES	YES	YES (-)	YES	CT	YES(-)	YES (-)	YES	+	M
McMahon 2014	YES	YES	YES	YES (-)	YES	CT	YES	YES	YES	+	H
Melberg 2016	YES	YES	YES	YES	YES	YES	YES	YES	YES	+	H
Mselle 2017	YES	YES	YES	YES (-)	YES	YES	YES	YES	YES	+/-	H
Morgan 2017	YES	YES	YES	YES(-)	YES (-)	YES	YES	YES	YES	+	H
Ochieng 2019	YES	YES	YES	YES (-)	YES	YES (-)	YES	YES	YES	+	H
Ongolly 2019	YES	YES	YES	YES	YES	CT	YES	CT	YES	+	H
Probandari 2017	YES	YES	YES	YES (-)	YES	YES	YES	YES (-)	YES	+	M
Sialubanje 2014	YES	YES	YES(-)	YES	YES	NO	YES	YES	YES	+	H
Sacks 2017	YES	YES	YES	YES (-)	YES	NO	YES	YES (-)	YES	+	M
Yakong 2010	YES	YES	YES	YES	YES	YES	YES	YES	YES	+	H
Yevoo 2018	YES	YES	YES	YES	YES	YES	YES	YES	YES	+	H
Zamawe 2015	YES	YES	YES	YES	YES	YES	YES	YES	YES	+	H

*CT: Cannot tell; +: significant value; +/-: some value; -: low value
Tool: Critical Appraisal Skills Programme (CASP) quality-assessment tool

Appendix 4. Assessment of confidence of qualitative findings: CERQual approach

Themes and subthemes	Contributing studies	Confidence in the Evidence*	Explanation of confidence in the evidence
**Women direct experience**			
**Health system's constraints:**			
Women, men and health providers reported lack of space, staff shortages, and long waiting times as major factors experienced during childbirth and deterring women from using PNC. Facility cleanliness was mentioned by women as a deterrent for accessing care.	Chen 2014 , Ganle 2015 , Mahiti 2015, Mselle 2017, Sialubanje 2014, Zamawe 2015,	Moderate	6 studies with minor methodological limitation. High relevance and coherence. Adequate data from 5 countries from Asia and Africa.
**D&A during previous contacts with health system:**			
Major complaints in this category were verbal abuse and condescension towards women. Many women reported being scolded or receiving derogatory comments during their previous contact with the clinical. The time and type of communication about postnatal care was also reported by many women. Many women expressed concerns about health workers inability to ensure privacy. Many women reported feeling neglect as they indicate that health workers sometimes delay services during their official work hours.	Dol 2019, Mselle 2017, Morgan 2017, Probandari 2017, Sacks 2017, Sialubanje 2014, Yevoo 2018, Yakong 2010	High	8 studies with moderate to minor methodological limitations. High relevance and coherence. Fairly thick data from 5 countries of Asia and Africa
**Women's expectations**			
**Internalised stigma:**			
Many of the papers reported that women would not seek postnatal care because they fear being embarrassed. These claims went from the embarrassment of giving birth to a child with poor health, to not having money to pay "a penalty" for using the services. In addition, many women reported not seeking care because they were embarrassed of not having proper clothing for the baby to wear. Fear of repercussion and denial of care was also mentioned in relation with the woman failing to follow health provider's recommendations from past visits .	Kane 2018, McMahon 2014, Morgan 2017, Melberg 2016, Ochieng 2019, Sacks 2017	High	6 studies with moderate to minor methodological limitation. High relevance and coherence. Fairly thick data from 7 countries from Africa.
**Beliefs and traditions**:			
Lack of culturally sensitive care acted as deterrent or delaying factor to PNC use. In this category it stands out the fear of medicalization and vaccination that still exists associated with Western medicine.	Dol 2019, Probandari 2017, Ochieng 2019	Low	3 studies with moderate to minor methodological limitations. High relevance and adequate coherence. Reasonable data from 3 countries in Asia and Africa.
**Women's expectations**			
**Male involvement and gender dynamics:**			
The role of father during pregnancy, childbirth and the postnatal period was often ambiguous. Maternity care was understood to be a woman's issue, deterring man's involvement. Traditional gender roles removed decision making power of the woman, affecting use of PNC. Some women referred that delays in care or certain practices from health care providers might have violent consequences when they return home.	Ganle 2015, Mselle 2017, Morgan 2017, Ongolly 2019	Moderate	4 studies with moderate to minor methodological limitations. High coherence. Reasonable data from 4 countries only in Africa.
**Family influence:**			
Women who live in the same house as their parents, parents in law or grandparents were more susceptible to allowing their opinion to influence their health seeking behaviour. Even if the advice provided by the family members contradicts the recommendation of health providers, women reported to feel oblige to go by what the family member says.	Dol 2019, Mselle 2017, Ochieng 2019, Probandari 2017	Low	4 studies with moderate to minor methodological limitations. Reasonable relevance and coherence. Reasonable data from 4 countries only in Africa.

* This approach considers four components: 1) Methodological limitation; 2) relevance to the review question; 3) Coherence and 4) adequacy of the data. The methodological limitation was assessed by the CASP tool in Appendix 3. The relevance refers to the extent to which the body of the data supporting the review finding is applicable to the context from the review question. The coherence is the assessment of how clear and cogent the fit is between the data from the primary studies and the review finding. Finally, adequacy is the degree of richness and quantity of data supporting the review finding.

Appendix 5. Summary of quantitative findings of the association between different domains of disrespect and abuse during childbirth and outcomes as reported in original article

Study	Outcome	Reported exposure domain*	Reported measures of effect*	Effect size (95% CI)	Adjustment variables
Creanga 2017	Maternal or neonatal healthcare services utilization	Perception that staff ensures patient's privacy (ref=no)	Adjusted Odds Ratio (95% CI)	1.43 (0.8;2.58)	Adjusted for socio-demographic characteristics (parity, religion, ethnicity, marital status, reading level, and household wealth), for women's perceptions of the quality of care at the closest health facility to their homes and for the time needed to reach this facility
		Perception that staff provides high quality services (ref=no)		1.54 (0.98;2.43)	
		Perception that facility is clean (ref=no)		1.24 (1.34;3.55)	
Bishanga 2019	Maternal healthcare services utilization	Experience any disrespect and abusive (ref=yes)	Adjusted Risk Ratio (95%CI)	1.23 (1.05;1.22)	Adjusted for region, mother's age, women's education, number of children, mode of delivery, cadre of provider attending the delivery, the type of health facility, and the number of ANC visits
		Offered choice of birth position (ref=no)		1.18 (1.02;2.23)	
		Offered opportunity to have a companion during labour and delivery (ref=no)		1.13 (0.97;1.84)	
		Perceiving facility to have good cleanliness (ref=no)		1.54 (1.54;3.02)	
	Neonatal healthcare service utilization	Experience any disrespect and abusive (ref=yes)		1.14 (1.02;1.92)	
		Offered choice of birth position (ref=no)		1.12 (0.93;1.84)	
		Offered opportunity to have a companion during labour and delivery (ref=no)		1.21 (1.02;1.69)	
		Perceiving facility to have good cleanliness (ref=no)		1.62 (1.70;3.71)	
de Sà 2016	Breastfeeding within an hour	Physical violence during delivery (ref= yes)	Adjusted Prevalence Ratio (95% CI)	0.96 (0.85;1.15)	Adjusted for maternal age, education, race, socioeconomic status, assistance to prenatal care, delivery care, children's health and characteristics (gender, birth weight, gestational age, Apgar score at 5 min)
		Verbal violence during delivery (ref=yes)		1.03 (0.92;1.15)	
		Companion at delivery (ref=yes)		0.95 (0.84;1.03)	
		Companion at postpartum (ref=yes)		1.03 (0.94;1.14)	
		Neglect during delivery (ref=yes)		0.98 (0.88;1.19)	
		Rooming-in (ref=yes)		0.28 (0.18;0.44)	
Silveira 2019	Postpartum depression [EPDS score>=13]	Any disrespect and abuse (ref=no)	Adjusted Odds Ratio (95% CI)	1.54 (1.15;2.05)	Adjusted for maternal education, family income, skin colour, age, parity, desire of pregnancy, marital status, father reaction when discovering pregnancy, pregnancy morbidities, deliver type and history of depression.
		Physical abuse (ref=no)		1.54(0.9;2.65)	
		Verbal abuse (ref=no)		1.58 (1.06;2.33)	
		Undesired procedures (ref=no)***		1.34(0.82;2.20)	
		Denial of care (ref=no)		1.48 (0.91;2.41)	
	Postpartum depression [EPDS score>=15]	Any disrespect and abuse (ref=no)		1.86 (1.32;2.63)	
		Physical abuse (ref=no)		2.28 (1.26;4.12)	
		Verbal abuse (ref=no)		1.69 (1.06;2.70)	
		Undesired procedures (ref=no)***		1.32 (0.71;2.46)	
		Denial of care (ref=no)		1.56 (0.86;2.80)	

CI= confidence interval
* Presented exactly as shown in the original article, without transformation.
***Included any procedure conducted against women's will or without explaining the need to conduct it, such as episiotomy or medication to induce labour
